# Role of the Immunogenic and Tolerogenic Subsets of Dendritic Cells in Multiple Sclerosis

**DOI:** 10.1155/2015/513295

**Published:** 2015-01-29

**Authors:** Zhong-Xiang Xie, Hong-Liang Zhang, Xiu-Juan Wu, Jie Zhu, Di-Hui Ma, Tao Jin

**Affiliations:** ^1^Neuroscience Center, Department of Neurology, The First Hospital of Jilin University, Changchun 130021, China; ^2^Department of Neurobiology, Care Sciences and Society, Karolinska Institute, 141 86 Stockholm, Sweden

## Abstract

Multiple sclerosis (MS) is an immune-mediated disorder in the central nervous system (CNS) characterized by inflammation and demyelination as well as axonal and neuronal degeneration. So far effective therapies to reverse the disease are still lacking; most therapeutic drugs can only ameliorate the symptoms or reduce the frequency of relapse. Dendritic cells (DCs) are professional antigen presenting cells (APCs) that are key players in both mediating immune responses and inducing immune tolerance. Increasing evidence indicates that DCs contribute to the pathogenesis of MS and might provide an avenue for therapeutic intervention. Here, we summarize the immunogenic and tolerogenic roles of DCs in MS and review medicinal drugs that may affect functions of DCs and have been applied in clinic for MS treatment. We also describe potential therapeutic molecules that can target DCs by inducing anti-inflammatory cytokines and inhibiting proinflammatory cytokines in MS.

## 1. Introduction

Multiple sclerosis (MS) is an autoimmune disease in the central nervous system (CNS) that is characterized by inflammation and demyelination as well as axonal and neuronal degeneration [1]. Plenty of immune cells participate in the pathogenesis of MS, which include dendritic cells (DCs), natural killer cells, B cells, and macrophages. DCs are professional antigen presenting cells (APCs) which are of great importance in mediating immune responses by providing signaling transduction for naive T cells to differentiate into myelin-reactive T cells. The latter are responsible for demyelination in CNS, one of the main pathological features of MS. To date, there has been no cure for MS. Current therapeutic strategies are focused on reducing the incidence of relapse and on alleviating the symptoms of the disease. Indeed, most of the therapeutic compounds and molecules at present are immune modulators or inhibitors which may have an effect on DCs. As DCs play an important role in immune tolerance, tolerogenic DCs may be induced to deal with MS relapses. Here, we summarize the effects of the different therapeutic compounds and molecules on DCs in MS. Specifically, we describe compounds that can both induce tolerogenic DCs and reduce MS occurrence and relapses. We also mention several potential therapies for MS that target DCs by inducing anti-inflammatory cytokines and inhibiting proinflammatory cytokine production.

## 2. Dendritic Cell Subsets and Biological Function

DCs are ubiquitous in the body. There are two major subsets of DCs: conventional DCs (cDCs; also known as myeloid dendritic cells (mDCs)) and plasmacytoid DCs (pDCs) [2], as shown in Table 1. In mouse, conventional DCs express both CD11c and MHCII and can be further subdivided into two major subsets based on the expression of CD8*α*: CD8*α* (+) DC and CD8*α* (−) DC [3, 4]. The former induces Th1 type responses while the latter drives Th2 type responses [5, 6]. However, human's cDCs are lack of expression of CD8*α* and are labeled based on other markers, namely, CD11c and HLA-DR. CD11c can be further subdivided into three subsets: CD1c+ (BDCA-1), CD141+ (BDCA-3), and CD16+DCs based on the expression of distinct cell surface markers [7]. CD16+DCs are considered to be a subset of both DCs and monocytes, because of their expressions of CD1c+ (BDCA-1) and CD141+ (BDCA-3) [8]. CD1c+DCs and CD141+DCs have been extensively studied for their unique gene expression profiles and special functions [9]. For example, CD141+DCs are located in human lymph nodes, bone marrow, tonsil, blood, and spleen [9, 10] with high expression of toll-like receptor 3 (TLR3) and IL-12p70 and IFN-*β* secretion [11]. Like their functional murine counterpart CD8*α*+DC subset, they induce Th1 responses and cross-present exogenous antigen [8]. CD1c+DCs, in turn, express most of the TLRs including the extracellular TLRs (TLR1, TLR2, TLR4, TLR5, and TLR6) and the endosomal TLRs (TLR3 and TLR8) [11]. When activated with TLR agonists, CD1c+DCs secrete high level of IL-8 but low level of TNF-*α*, IL-6, CCL3 (MIP-1*α*), and CCL4 (MIP-1*β*), indicating the strong inflammatory activity [7]. When CD1c+DCs were stimulated with* Escherichia coli*, high levels of anti-inflammatory cytokine IL-10 and regulatory molecules indoleamine 2, 3-dioxygenase (IDO) as well as soluble CD25 were produced. Moreover,* E. coli*-activated CD1c+DCs suppressed T-cell proliferation in an IL-10-dependent manner [12].

pDCs can be found in both lymphoid and nonlymphoid organs. Unlike cDCs, pDCs do not express CD11c, but HLA-DR, CD123, BDCA-2(CD303), and BDCA-4 (CD304), which can be used as markers to isolate pDCs [13]. Through TLR7 and TLR9, pDCs could recognize viral DNA or RNA and secrete large amounts of type I interferons (IFN) [14]. pDCs can also secrete moderate amounts of TNF-*α* and IL-6 upon viral stimulation. The former serve to either promote the maturation of pDCs in an autocrine manner or mediate immune response while the latter mediate immune responses by inducing plasma cell differentiation and immunoglobulin secretion [15, 16]. Some researchers divide human pDCs into two subsets: pDC1 and pDC2 [17]. The pDC1 expresses high level of CD123 and low level of CD86 and TLR2; in addition, it secretes IFN-*α* and induces IL-10 producing T cells [17]. The pDC2, in turn, is characterized by low CD123 expression and a high level of CD86 and TLR2 [17]. Moreover, they are the main source of plasma IL-6 and IL-12 and mediate the differentiation of naive T cells into Th17 cells [17].

Under the steady state, pDCs display an immature phenotype with a very limited capability to induce naive T cell activation [18]. Upon activated through either IL-3 or virus CpG oligo nucleotides, pDCs differentiate into mature DCs and can form stable connections with T cells [19], which significantly enhance their capacity to activate these lymphocytes [15]. pDCs are also involved in immune tolerance with the potential to induce T regulatory cells (Tregs) and upregulate expression of IDO when they are exposed to a TLR9 agonist and activated [20]. Specifically, mature pDCs upregulate the expression of inducible costimulator ligand (ICOS-L) and induce differentiation of naive T cells into IL-10 secreting Tregs [21].

Tolerogenic DCs are generally viewed as a steady state semimature DCs which can express costimulatory molecules but did not produce proinflammatory cytokines. They can efficiently induce Tregs instead of inducing Th1/Th17 responses [22]. Both tolerogenic DCs and immature DCs would induce Tregs, but the difference between tolerogenic DCs and immature DCs is that the former are more stable than the latter.* In vivo*, after being exposed to the inflammatory microenvironment tolerogenic DCs will change much less than immature DCs [23]. Tolerogenic DC would be induced* in vitro* by modulating DCs with cytokines and drugs (e.g., IL-10 and VD3). Recently, researchers have found a new subset of tolerogenic DC: DC-10. In peripheral blood, DC-10 is characterized by HLA-G expression and IL-10 secretion and is essential in promoting and maintaining tolerance via induction of Tregs [24]. This discovery provides us new perspective for autoimmune disease treatment.

**Figure 1:**
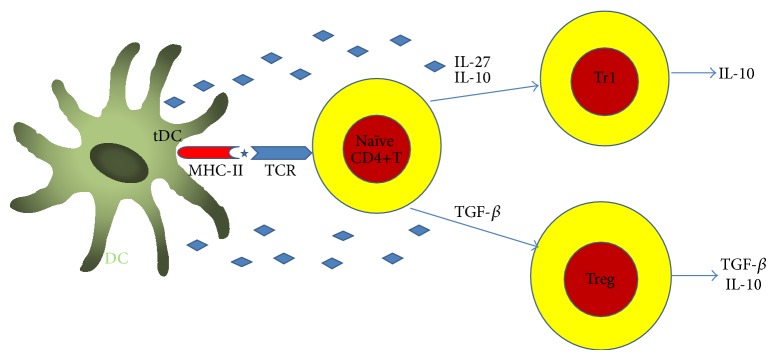
Tolerogenic dendritic cells play their tolerogenic role through promote regulatory T cells differentiation. The tolerogenic DCs regulate autoreactive T cells by inducing anergy, apoptosis, phenotypically skewing, and/or Treg cells or tolerogenic DCs can be induced through the induction of T regulatory cells, such as Tr1 and CD4+, CD25+, and Foxp3 cells.

Tolerogenic DCs bear several characteristics: low expressions of costimulatory molecules (CD80, CD86, and CD40) and high levels of surface molecules (PDL1 and CD95L) which are involved in T-cell inhibition [25]. Low levels of costimulatory molecules contribute to T cell anergy, as T cells cannot receive the secondary signals for stimulation. However, the high levels of suppressive cytokines, such as IL-10, suppress the immune response. Alternatively, tolerogenic DCs can induce the development of CD4+FoxP3+ Tregs and type 1 regulatory T cells (Tr1), which can secrete both IL-10 and TGF-*β*. Tregs in the CNS can downregulate the immune response by secreting IL-10, thereby inducing anergy or inhibiting the T cell effector response [26]. As previously described, tolerogenic DCs express IDO which catalyzes tryptophan either resulting in depletion of the microenvironment, or consequently inhibiting the proliferation of T-cells [27]. Taken together, tolerogenic DCs regulate autoreactive T cells by inducing anergy, apoptosis, phenotypically skewing, and/or Treg cells [28, 29] (Figure 1). The therapeutic potentials of tolerogenic DCs in EAE, an animal model of MS, have been further reviewed [30].

## 3. Dendritic Cells in the Central Nervous System

As previously described, DCs are ubiquitous in the body including the CNS. Although it is difficult to find DCs in healthy CNS parenchyma due to the immune privilege, DCs have been isolated from vascular-rich compartment, that is, the choroid plexus and the meninges [31, 32]. In addition, cDCs and pDCs can also be isolated from the cerebrospinal fluid (CSF) of healthy individuals [33]. Interestingly, a recent study reported that DCs can also be found in the brain parenchyma near the vessels (juxtavascular) [34], which challenged the traditional concept that DCs reside only in the perivascular space. However, the source of the DCs in the CNS remains unclear thus far. Some studies* in vitro* showed that the resident microglia in the CNS could differentiate into DCs in the presence of granulocyte-macrophage colony-stimulating factor (GM-CSF) [35, 36]. Cytokines including IL-6 and TNF-*α* can induce DC differentiation in the CNS as well [37]. Moreover, DCs in the brain may originate from the periphery, and it has been found that Fms-like tyrosine kinase 3 ligand (Flt3L) can induce the proliferation of DCs [38] and recruit pDCs to the brain parenchyma [39]. Using the criterion of Flt3-dependent development, Anandasabapathy and colleagues found that DCs in the meninges and choroid plexus of a healthy mouse brain exhibited differentiation and antigen presenting program similar to spleen DCs while being distinct from microglia, indicating that DCs in a healthy brain possibly arise from pre-DCs which enter the brain perivascular region [40]. Besides, the juxtavascular locations of DCs [34] also provide indirect evidence suggesting the brains DCs are not derived from the brain tissue but from a vascular source.

## 4. Role of Dendritic Cells in MS

### 4.1. MS Introduction

**Figure 2:**
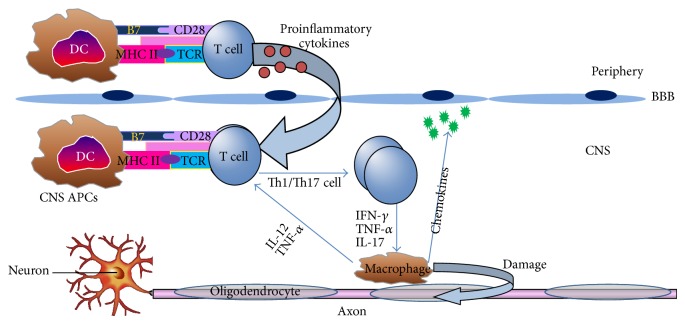
Role of dendritic cells (DCs) in the pathogenesis of multiple sclerosis (MS)/experimental autoimmune encephalomyelitis (EAE). As professional antigen-presenting cells (APCs), DCs in the periphery could activate the T cells upon pathological stimulation resulting in secreting proinflammatory cytokines, aiding their entry through the endothelial blood-brain barrier (BBB) to the CNS; then these myelin-reactive T cells are reactivated upon encounter with resident APCs including DCs which present myelin-derived epitopes. Subsequently, these perivascular T cells will secrete proinflammatory cytokines which result in recruitment of other inflammatory cells. Consequently, this will lead to demyelination of axons accounting for the sensory and motor deficits of MS.

Multiple sclerosis (MS) is a multifactorial autoimmune disorder of the CNS characterized by chronic inflammatory demyelination with hallmark of focal infiltration and accumulation of immune cells resulted in the subsequent damage to the myelin and axons [41]. Based on the clinical data and the histopathological studies, four clinical subtypes of MS have been identified, including relapsing-remitting MS (RRMS), primary progressive MS (PPMS), secondary progressive MS (SPMS), and progressive relapsing MS (PRMS), among which the RRMS accounts for approximately 85% of all MS cases [41]. Demyelination and axonal loss are two characteristics of MS. Demyelination and axonal loss are closely influenced although it remains controversial regarding the causal relationship. Currently, “the inside-out model” and “the outside-in model” are two competing hypotheses of MS etiology. The outside-in model refers to demyelination caused axonal loss and neurodegeneration, while the inside-out model believes that neural and axonal damage is caused by demyelination [42]. Experimental autoimmune encephalomyelitis (EAE) is the traditional animal model of MS. In EAE, mice were immunized with myelin specific antigen, the antibodies and autoreactive T cells are against myelin itself or oligodendrocyte, and axons damage developed from myelin (outside) to axons (inside) [43]. Mice immunized with neurofilament light (NF-L) protein bear axonal degeneration and gray matter inflammation with mild demyelination, which is representative of the inside-out model [44]. Besides, Theiler's murine encephalomyelitis virus- (TMEV-) induced demyelination disease (TMEV-IDD) is a viral model for MS. In TMEV infection the lesion developing from the axons (inside) to the myelin (outside) is also an inside-out model [43]. In patients with MS, evidence exists to support both “the inside-out model” and “the outside-in model” [43]. Some neurologists hence argue that MS may be an unrecognized primary degenerative disorder and consider noninflammatory PPMS as the real MS, with inflammatory forms reflecting secondary host's aberrant immune responses [45]. Although MS was considered to be predominantly a T cell-mediated disease, emerging evidence indicates that DCs play a crucial role in the pathology of MS. As natural immunomodulators and professional APCs, DCs serve as an orchestrator to preserve the balance between immunity and tolerance due to their unique ability to stimulate naive T cells. Upon pathological activation by DCs in the periphery, the myelin-reactive T cells secrete proinflammatory cytokines which aid their entry through the endothelial blood-brain barrier (BBB) to the CNS. These myelin-reactive T cells are then reactivated upon encounter of resident APCs including DCs, presenting myelin-derived epitopes. Subsequently, these perivascular T cells will secrete proinflammatory cytokines which recruit other inflammatory cells. Consequently, this will lead to demyelination of nerves accounting for the sensory and motor deficits of MS (Figure 2).

As professional APCs, DCs can play both immunogenic and tolerogenic roles. DCs are involved in the pathogenesis of MS at both stages of initiation and development [46]. Under steady state, DCs display an immature phenotype characterized by expression of low levels of costimulatory molecules, which are involved in the processing and presenting antigens to T cells inducing peripheral tolerance. Upon maturation, the expression of costimulatory molecules and cytokines on DCs is upregulated. These changes enhance their immunogenicity and enable their capacity to induce naive T cells to differentiate into different types [47]. It is noteworthy that some mature DCs can also induce immune tolerance.

### 4.2. DCs in Animal Model of MS

**Figure 3:**
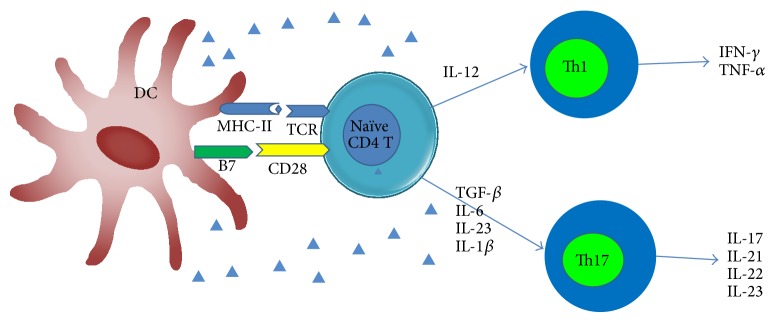
Dendritic cells (DCs) promote the differentiation of Th1 and Th17 cells. Mature DCs could induce the differentiation of naive CD4+ T cells into different types of T helper (Th) cells. Both Th1 and Th17 cells play pathogenic roles in the disease progression of MS mainly through their cytokines.

Autoreactive CD4+ T cells, including Th1 and Th17 cells, are essential in the pathogenesis of experimental autoimmune encephalomyelitis (EAE). Specifically, the autoreactive CD4+ T cells in the CNS can damage the neuronal axon leading to MS [48] (Figure 3). In peripheral lymphoid nodes, DCs can activate self-antigen specific naive CD4+ T cells and subsequently promote them to differentiate into Th1 and Th17 effector cells [47]. At this point, DCs in the perivascular space of the CNS can reactivate autoreactive T cells in the vicinity and facilitate their infiltration into the parenchyma of the CNS [49]. cDCs are sufficient to activate encephalitogenic T cells in both EAE and TMEV-IDD [50, 51]. However, depletion of cDCs did not affect the activation of encephalitogenic Th1 and Th17 cells in EAE [22]. Taken together, these data suggest that although DCs are sufficient to prime EAE, other APCs were also needed.

Tregs have a crucial tolerogenic effect on EAE. Depletion of Tregs in the brain worsened the severity of EAE, while passive transfer of CNS derived Tregs seemed protective [52]. Mice with EAE showed upregulated levels of Treg cells and improved disease prognosis after being injected with DCs, which expressed myelin oligodendrocyte glycoprotein (MOG) and TNF-related apoptosis-inducing ligand (TRAIL) on cell surface [53]. Of note is that depletion of DCs in mice would worsen EAE. DCs are responsible for the upregulation of PD-1 on antigen-specific T cells and subsequently induce the* de novo *conversion of Treg cells from naive T cells during immune responses [54]. PD-1 and PD-L1 mRNA expression were elevated on BMDCs from TMEV-IDD mice. Moreover, TMEV-IDD mice treated with PD-1 antibody exhibit more severe clinical presentation, indicating PD-1 pathway plays a pivotal regulatory role in the development of TMEV-IDD [55].

pDCs have been evidenced to have an immunosuppressive role in EAE. During the acute and relapsing phases of EAE, depletion of pDCs enhanced pathogenic CNS CD4+ T cell activation as well as IL-17 and IFN-*γ* production [56]. DCs can also function as APCs to promote the expansion of myelin-Ag-specific natural Tregs [57]. These natural Tregs suppress the autoimmune T cell response and thus confer a natural protection against EAE [57]. In sum, DCs play both pathogenic and regulatory role in MS model.

### 4.3. DCs Phenotype and Function Change in MS

DCs, including cDCs and pDCs, were abundant in the inflamed CNS lesions and CSF of patients with MS. Additionally, circulating DCs, which secrete high levels of proinflammatory cytokines, are elevated in MS [58]. Thus, levels of DCs in the CSF of MS patients are considered to be a clinically relevant marker for MS [59]. At the early stage of MS, the number of DCs in the demyelinating lesions of CNS is increased [60]. The expression of CD40 on cDCs from both RRMS and SPMS is higher compared with controls [61], while cDCs of patients with PPMS express lower levels of costimulatory molecules like CD80 and CD86 [62]. In patients with SPMS, cDCs showed a proinflammatory profile in which the expression of CD80 is elevated, PD-L1 is decreased, and the secretion of proinflammatory cytokines including IL-12 and TNF-*α* is also elevated [61]. Further investigations showed that cDCs from SPMS only induced a polarized Th1 response, while cDCs from RRMS induced higher levels of Th1 (IFN-*γ*, TNF-*α*) and Th2 (IL-4, IL-13) cytokines compared with controls [61]. cDCs of patients with MS may produce elevated amounts of IL-23 compared with healthy controls [63]. Besides, the expression of chemokine (C-C motif) receptor 5 (CCR5) on cDCs was upregulated in both blood and CSF of patients with MS [33]. Interestingly, CCR5 ligands chemokine (C-C motif) ligand 3 (CCL3) and CCL5 are increased in plaques of patients with MS, providing a possible explanation that recruitment of cDCs to the inflamed CNS may be related to this pathway [64]. In summary, cDCs of patients with MS manifest a proinflammatory phenotype which would be critical in the pathogenesis of MS.

In patients with MS, pDCs can be found in CSF, in lesion plagues, and also in leptomeninges [33, 65]. pDCs would increase in CSF of untreated patients during relapse and this may be explained by either a virus infection or a downregulatory process [66]. In MS patients, the phenotype and function of pDCs would be affected. The expression of CD86 and 4-1BBL was significantly lower on pDCs from MS patients than from controls [67]. And when stimulated with CD40L or IL-3, pDCs from MS patients showed impaired maturation ability; the upregulation of CD86, 4-1BBL, CD40, and CD83 was inhibited [67]. In RRMS patients, the expression of CCR7 on pDCs was upregulated compared with healthy controls [68]. The pDC1 expresses a high level of CD123 and a low level of CD86 and TLR2; in addition, it secretes IFN-*α* and induces IL-10 producing T cells [17]. The pDC2, in turn, is characterized by low CD123 expression and high levels of CD86 and TLR2 [17]. Moreover, pDC2 are the main source of plasma IL-6 and IL-12 and mediates the differentiation of naive T cells into Th17 cells [17]. pDCs in MS have a lower pDC1/pDC2 ratio in the peripheral blood, denoting that pDCs in MS patients have a proinflammatory profile [17].

Beside the phenotype change of pDCs in MS, the cytokines secretion of pDCs was also affected. In patients with MS, upon stimulation with CpG, pDCs from PBMC have a significantly lower IFN-*α* secretion than in controls [67]. Moreover, pDCs from MS lost the capacity to induce proliferation and IFN-*γ* secretion of allogeneic lymphocytes [67].

## 5. Therapeutic Options for MS

So far there has been no cure for MS. Current therapeutic compounds mainly lessen the symptoms and reduce the frequency of relapse. The effects of therapeutic compounds on MS by affecting DCs are listed in Table 2.

### 5.1. Corticosteroids

Corticosteroids are most effective during the acute phase of MS relapses. Intravenous methylprednisolone (MP) treatment accelerates the clinical recovery in acute MS relapse, but the mechanism or the effect on DCs is not well understood. After a short-term treatment with IVMP, pDCs and cDCs in the peripheral blood significantly decreased, while Tregs are increased, indicating that the immunosuppressive effect of MP may be related to decreased numbers of circulating DCs and increased Tregs [69]. Moreover, circulating pDCs decreased after corticosteroid therapy and then are increased again during the remission period, that is, 30 days after treatment was completed [70]. Therefore, DCs may exert their therapeutic effects on partly through decreasing the number of pathogenic DCs.

### 5.2. Interferon-*β*

Interferon- (IFN-) *β* is an immunomodulatory cytokine and is used as another therapeutic compound against RRMS. The mechanism, however, remains incompletely clear. The effect of IFN-*β* on DCs is very complex. IFN-*β* could affect the secretion of cytokines from DCs. It has been found that DCs from MS patients secrete low levels of IL-12p70 and high levels of IL-10 after IFN-*β* therapy [71]. And IFN-*β* inhibits IL-12p70 secretion by mature DCs but enhances IL-12p70 secretion by immature DCs. IFN-*β* can as well counteract the IL-12-enhancing effect of IFN-*γ* on DCs irrespective of their maturation status [72]. Zhang et al. found that IFN-*β* plays a crucial role in inhibiting Th17 response through affecting DCs, because human-derived DCs treated with IFN-*β* may upregulate the expression of TLR7, reduce IL-1*β* and IL-23, and increase IL-27, all of which could inhibit Th17 differentiation [73]. Moreover, IFN-*β* may inhibit IL-1*β*, IL-23, and TGF-*β* and induce secretion of IL-27, IL-12, and IL-10, all of which contribute to the suppression of Th17 cell differentiation. In sum, IFN-*β* has a therapeutic value for RRMS [41]. Among other cytokines, IL-27 plays a unique role in IFN-*β* treatment for MS. Sweeney and colleagues found that IFN-*β* induced IL-27 both* in vitro* and* in vivo*. Additionally, IL-27 induced by IFN-*β* was associated with response to IFN-*β* therapy in MS patients. This finding indicates that the therapeutic role of IFN-*β* in MS is partly mediated through IL-27 [74].

IFN-*β* may also affect the maturation of DCs in MS. The* in vitro* experiments showed that when monocytes derived DCs at different stages of maturity were stimulated with IFN-*β*, the development of DCs was inhibited only at the early stage of maturity [75]. In MS patients treated with IFN-*β*, the function and phenotype of pDCs were evaluated before and during the treatment. And the results exhibited that the expression of MHC-II and BDCA-2 molecules were decreased, while the expression of costimulatory molecules such as CD83 and B7H1 were upregulated [65]. B7H1 (PD-L1), a member of B7 family proteins with the ability to inhibit CD4 T-cell activation expressed on DCs, was also upregulated* in vitro* in MS patients [76]. Besides, IFN-*β* also has the capacity to inhibit DC migration via inhibiting the expression of CCR7 and matrix metalloproteinase- (MMP-) 9 proteins in mature DCs, which consequently reduces DCs migratory capacity [77] and impaired the antigen presentation role of DCs, and enhancing their anti-inflammatory abilities.

IFN-*β* therapy in MS can change the subtype and numbers of DCs. RRMS patients treated with IFN-*β* were found with decreased number of circulating DCs [62]. MS patients were even found to be in a reversed ratio of pDC1/pDC2 in peripheral blood (4.4 : 1 in healthy controls versus 0.69 : 1 in MS patients). In addition, IFN-*β* treatment increased the pDC2 cells* in vivo*, which reconstituted the disturbed balance [17]. Apart from its influence on the subset and quantity of pDCs, IFN-*β* could reduce the level of processed TLR9 protein of pDCs from MS patients. This would decrease activation of pDCs by viral pathogens and might hinder the relapse of MS [17]. Besides, IFN-*β* upregulate the expression of glucocorticoid-induced tumor necrosis factor receptor ligand (GITRL) on DCs, and the increased GITRL consequently enhances the proliferation of Tregs through the ligation of GITR [78]. Meanwhile, IFN-*β* depresses the expression of CTLA-4 on Treg cells, thus promoting Treg cells stimulation [78]. Interestingly, a recent study showed that there were 60 abnormal genes expressed on pDCs in MS patients, while the expression pattern was normalized after IFN-*β* therapy [79].

In conclusion, IFN-*β* induce anti-inflammatory cytokines secretion and inhibit proinflammatory cytokines secretion by DCs. IFN-*β* decrease the costimulatory molecule and chemokine molecules expression on DCs; through this way IFN-*β* may inhibit T cell activation and release the infiltration of T cells in CNS. Besides, IFN-*β* upregulate the expression of GITAL on DCs which would induce the proliferation of Tregs.

### 5.3. Glatiramer Acetate

Glatiramer acetate (GA), which is a mixture of synthetic polypeptides comprising four amino acids, has been widely used for treating RRMS. GA is an analog of myelin basic protein (MBP) with the potential to compete with MBP for MHC binding. GA functions by inducing GA-specific T cells which shift T cell balance from a dominant proinflammatory phenotype (Th1/Th17) to an anti-inflammatory phenotype (Th2/Treg) [80]. The therapeutic potential of GA in MS is related to its effects on DCs. GA can modulate the secretion of cytokines by DCs.* In vitro*, GA reduces IL-12 secretion from DCs in MS patients [81]. Vieira et al. reported that GA inhibited the production of Th1 polarizing factor IL-12p70 and induces the production of anti-inflammatory cytokine IL-10 from DCs [82]. Moreover, GA interfered with expression of osteopontin, IL-17, and RAR-related orphan receptor gamma (ROR*γ*t) in DCs of mice with EAE; therefore, biased DCs shift into an anti-inflammatory phenotype [83]. GA not only biased DCs towards the anti-inflammatory phenotype, but also affected their penetration through the BBB during neuroinflammation in EAE. GA could suppress the expression of molecules that affect the BBB penetration during neuroinflammation [83].

GA reduced the HLA-DR expression of DCs both* in vitro* and* in vivo*. [84]. pDCs from MS patients exhibited a significantly lower level of CD86 and 4-1BBL, and GA treatment partially restored the phenotype and function of pDCs in MS [67]. Additionally, MS patients treated with GA had lower levels of CD40 on DCs, which was associated with a lower risk of relapse in MS [85]. Besides, GA can enhance NK cells lysis of both immature DCs and mature DCs. CD86 and NKp30 are important for NK cell lysis of immature DCs, whereas CD80, CD83, HLA-DR, and HLA-I are important for lysis of mature DCs [86].

In summary, GA biased DCs toward anti-inflammatory phenotype and GA inhibit CD40 expression on DCs and thus lower the relapse risk of MS. The enhancement of NK lysis of DCs by GA may have an indirect effect for its therapeutic role on MS.

### 5.4. Natalizumab

Natalizumab is a monoclonal antibody for treating MS. It could block the very late antigen 4 (VLA-4), which is widely expressed on leukocytes and associated with the infiltration of leukocytes into the CNS. Natalizumab is effective against a number of APCs in the CNS. For example, the expression of MHC-II molecules and the number of CD209+DCs are significantly decreased in cerebral perivascular space of the natalizumab treated MS patients [87]. Natalizumab therapy may result in lower expression of VLA-4 on both pDCs and cDCs of MS patients. Besides,* in vitro* coculture experiments showed that natalizumab not only downregulated the expression of VLA-4, but also reduced the ability of DCs in stimulating the antigen-specific T-lymphocyte response [88]. Above all, although natalizumab mainly limits the infiltration of lymphocytes within CNS, its capacity to reduce VLA-4 on DCs may limit DCs infiltration in CNS and this capacity would be of utmost importance in the treatment of MS.

### 5.5. Fingolimod

Fingolimod (FTY720), a sphingosine analog, is the first oral drug for treating RRMS, which was approved by the US Food and Drug Administration in September 2010 [89, 90].* In vivo*, the drug binds to four out of the five sphingosine-1-phosphate (S1P) receptors after phosphorylation (FTY720-p) [91]. After the combination, FTY720 downmodulates S1P receptor expression on lymphocytes, slows the outflow of lymphocytes from secondary lymph organs and limits lymphocytes infiltration within the CNS [92].

*In vitro*, DCs treated with FTY720/FTY720-P impaired the chemotaxis and immunostimulatory capacity of DCs [92]. IL-12 secretion was reduced while IL-10 production was increased when mature DCs were treated with FTY720/FTY720-P. Similarly, T cells cultured in the presence of FTY720 or FTY720-P treated DCs showed an altered cytokine production profile which indicated a shift from Th1 toward Th2 differentiation [92].* In vivo*, FTY-720 modulates the traffic of DCs by reducing their capacity of migration to transendothelial [93]. A study indicated that FTY720 inhibited IL-12p70 secretion by DCs and macrophages while increasing IL-10 production in DCs [94]. Briefly, FTY720 may exert its immunosuppressive roles partly by modulation of DC trafficking. Although FTY720 impaired the chemotaxis and immunostimulatory capacity of DCs* in vitro*, it remains to study to what extent DCs are influenced by FTY720 treatment.

### 5.6. Immunosuppressive Drugs in MS Treatment

Several immunosuppressive drugs have been approved for treating MS patients, such as mitoxantrone and azathioprine. Some of these drugs play their immunosuppressive role through influencing the functions of DCs.

#### 5.6.1. Mitoxantrone

Mitoxantrone was initially used as an antineoplastic agent. In the United States, it has been approved for treating MS, including secondary-progressive MS, progressive-relapsing MS, and worsening RRMS [95].* In vitro*, mitoxantrone at low concentrations reduces the antigen presenting capability of DCs and apoptosis of DCs, whereas at higher concentrations it causes cell lysis [96]. Mitoxantrone exerts its cytotoxic and immunomodulatory effects on microglia in CNS. It can induce apoptosis of microglia by upregulating IL-10 and downregulating IL-23p19 secretion of microglia [97]. However, the specific effect of mitoxantrone on DCs remains to be elucidated.

#### 5.6.2. Azathioprine

Azathioprine (Aza) is another immunosuppressive drug that is commonly used in organ transplantation. It can also be used to treat autoimmune disorders such as MS. 6-Mercaptopurine (6-MP) is the active form of Aza. 6-MP is able to inhibit DC activation and induce the differentiation of DCs into a less immunogenic phenotype* in vitro*. Moreover, 6-MP significantly reduced the secretion of IL-23 and the expression of CCR7 on DCs while increasing the expression of IL-10 [98]. These findings indicate that Aza has a therapeutic role in MS via both immunosuppressive and immunomodulatory pathways.

In conclusion, immunosuppressive drugs including mitoxantrone and azathioprine would affect the function of DCs* in vitro*, but* in vivo* it remains to be studied to what extent DCs are influenced by this treatment.

### 5.7. New Drugs

Several new drugs show potential therapeutic effects on MS. Some of the drugs have been approved for the treatment of MS, while the others are still under clinical trials. Here, we summarize drugs that have direct or indirect effects on DCs.

#### 5.7.1. Teriflunomide

Teriflunomide is a newly approved oral drug for RRMS [99]. As a new oral medicine for MS, teriflunomide can reduce relapses and slow disability progression and its side effects are mild and transitory [100]. What is more, recently studies showed that teriflunomide has beneficial effects for patients with early MS [101]. Teriflunomide is an inhibitor of dihydro-orotate dehydrogenase (DHODH), which inhibits the proliferation of stimulated T and B cells, decreasing the number of lymphocytes infiltration within CNS [102].* In vitro*, teriflunomide decreases the secretion of proinflammatory cytokines IL-6, IL-8 and monocyte chemotactic protein-1 (MCP-1) in activated PBMC [103]. There was also research demonstrating that teriflunomide impaired Th1 differentiation and induce Th2 differentiation [104]. Teriflunomide did not impair LPS-induced maturation of DC, and the ability of matured DC to induce allogenic T cell responses was not affected [105]. Further study about teriflunomide on DC should be conducted in the future.

#### 5.7.2. Dimethyl Fumarate (DMF)

Dimethyl fumarate (DMF) is the third oral drug used for RRMS; it was approved by FDA in March, 2013 [106]. DMF and its primary metabolite monomethyl fumarate (MMF) have neuroprotective effects. DMF and MMF protect neurons and astrocytes against oxidative stress-induced cellular injury and loss; they exert the role mainly through upregulation of nuclear factor- (erythroid-derived 2-) like (Nrf2) dependent antioxidant response [107]. DMF affect the function of DCs. Through suppression of both NF-*κ*B and extracellular signal-regulated kinases 1 and 2 (ERK1/2) and mitogen stress-activated kinase 1 (MSK1), DMF inhibit DCs maturation and Th1 and Th17 differentiation [108]. Moreover, DMF treatment in human induces IL-4 producing Th2 cells and generates DCs that produce IL-10 instead of IL-12 and IL-23 [109]. In mice, DMF also generates such DCs that may induce Th2 cell differentiation* in vitro *and protect mice from EAE* in vivo*. The underlying mechanism has been explained as DMF resulting in glutathione (GSH) depletion and HO-1 induction phosphorylation. HO-1 prevents transcription of the IL-23p19, whereas signal transducers and activators of transcription (STAT) 1 inactivation inhibit transcription of the IL-12p35 [109]. This research indicated that DMF may play an important part in inhibiting Th1 and Th17, which are critical in the pathologies of MS.

#### 5.7.3. Laquinimod

Laquinimod is a novel oral drug that is under evaluation for the treatment for RRMS. Laquinimod exerts its immunomodulatory role through multiple ways. In mice, laquinimod inhibits peripheral proinflammatory T cells into CNS [110].* In vitro*, laquinimod decreases the secretion of proinflammatory cytokines and increases the secretion of anti-inflammatory cytokines from PBMC [110]. Jolivel et al. exhibited that the beneficial effect of laquinimod for MS mainly mediated by DCs. The authors found that human monocyte-derived matured DCs treated with laquinimod had reduced capacity to induce CD4+ T cell proliferation and proinflammatory cytokines secretion [111]. What is more, chemokine productions by both murine and human matured DCs were reduced when DCs were treated with laquinimod [111]. In laquinimod-treated patients, the chemokine and cytokine secretions were reduced in CD1c+ matured DCs, and the number of conventional CD1c+ and plasmacytoid CD303+DCs was decreased within peripheral blood mononuclear cells [111]. Moreover, in laquinimod treated DCs, expression of CD86 was inhibited. The authors believed that the inhibition of the NF-*κ*B pathway was responsible for the changes of dendritic cell maturation and functions [111]. A placebo-controlled phase III study showed that laquinimod treatment reduced disability progression and had a modest effect on annualized relapse rate [112]. A recent phase III study showed that laquinimod may reduce (at least in the initial phase of treatment) some of the more destructive pathological processes in patients with RRMS [113]. Another study showed that although once-daily oral laquinimod resulted in statistically nonsignificant reductions in ARR and disability progression, there were significant reductions in brain atrophy versus placebo [114].

#### 5.7.4. Daclizumab

Daclizumab, which blocks the interaction of CD25 with IL-2 and has been approved for renal transplant rejection, is a mAb specific for CD25 (*α* subunit of IL-2 receptor) [115]. Subcutaneous daclizumab high-yield process (HYP) administered every 4 weeks led to clinically important effects on MS disease activity during 1 year of treatment [116]. The mechanism of action of daclizumab for MS has been summarized in a recently published review [117]. Multiple immune cells are affected by daclizumab including natural killing cells (NK cells) and DCs. Human monocyte-derived DCs stimulated with LPS would induce the expression of CD25 on DCs [118]. LPS-matured DCs treated with daclizumab would change the cytokines secretion by DCs [118]. Proinflammatory cytokines such as IL-12, IL-1, TNF-*α*, IL-6, and IFN-*γ* production decreased and anti-inflammatory cytokines IL-10 increased [118]. Besides, the ability of DCs to prime allogenic T cells diminished, while the upregulation of costimulatory surface markers on DCs induced upon LPS stimulation was not affected [118]. Activated DCs could express IL-2R*α* subunit and secrete IL-2; IL-2R*α* subunit expressed on DCs captures IL-2 and forms a complex with the IL-2R*β* and *γ*c subunits expressed on the T-cell surface [117, 119]. Daclizumab also blocks trans-presentation of IL-2 and inhibit T cell activation and proliferation [117, 120]. Above all, daclizumab exerts its therapeutic role partly via converting DCs functions toward a tolerogenic profile.

#### 5.7.5. Alemtuzumab

Alemtuzumab is a mAb against CD52. CD52 is a surface molecule on T and B lymphocytes, NK cells, DCs, and most monocytes [121]. Alemtuzumab can deplete CD52 bearing cells and has been approved for the treatment of chronic B cell lymphocytic leukemia. Alemtuzumab can also improve relapse rate versus interferon beta-1a in patients with MS who were treatment-naive (CAMMS223 and CARE-MS I) or had relapsed on prior therapy (CARE-MS II), to reduce sustained accumulation of disability (CAMMS223 and CARE-MS II) [122]. Although alemtuzumab would cause some side effects such as serious infections, infusion-associated reactions, or even autoimmune events, safety monitoring program allowed for early detection and management of autoimmune events [122]. In US and Europe alemtuzumab has been submitted for licensing in RRMS. In May, 2014, the UK National Institute of Health and Care Excellence (NICE) recommended alemtuzumab as an option for the treatment of RRMS [123]. So far, effects of alemtuzumab on RRMS have not been found associated with DCs, while in previous studies alemtuzumab did affect the function of DCs. It is believed that alemtuzumab may deplete monocyte-derived DCs and its precursors [124]. CD52 is expressed on peripheral blood DCs but not on tissue DCs. Administration of alemtuzumab to patients with lymphoproliferative disorders resulted in circulating DCs reduction [125]. Analysis of monocyte-derived DCs* in vitro* showed the activation-induced maturation with lipopolysaccharide was lost [125]. Not only did alemtuzumab affect the quantity of DCs, but the phenotype of DCs was also changed. Alemtuzumab in patient with renal transplant caused reduction of the total number of peripheral DCs and a significant shift from myeloid to plasmacytoid DC subsets (mDC/pDC ratio) [126]. In sum, alemtuzumab depletes DCs which may be one of its main mechanisms in the treatment of RRMS.

#### 5.7.6. Secukinumab

Secukinumab is a mAb against IL-17A, also called SECU or AIN457. A phase II clinical study has been conducted to determine the efficacy and tolerance of AIN457, which revealed that AIN457 appeared to be superior to placebo and a majority of patients with RRMS tolerated it well [127, 128].

#### 5.7.7. MOR103

MOR103 is a mAb that may neutralize GM-CSF. GM-CSF stimulates activation, maturation, and differentiation of macrophages, monocytes, neutrophils, eosinophils, DCs, and microglia [128]. In EAE, blockade of GM-CSF led to reduced microglial activation [129]. A randomized, double-blind, placebo-controlled phase Ib study to evaluate the safety and pharmacokinetics of MOR103 has been completed. The results are accessible online while conclusions are yet to be reached (https://clinicaltrials.gov/ct2/show/results/NCT01517282?term=NCT01517282&rank=1&sect=X40156).

#### 5.7.8. Anti-IL12/23p40 Antibodies (ABT-847)

Both IL-12 and IL-23 are proinflammatory cytokines secreted by APCs. IL-12 and IL-23 have the common subunit IL-12/23p40. Anti-IL-12/23p40 antibody significantly ameliorated EAE in rodents [130, 131] and non-human primates [132, 133]. Unfortunately, clinical trials in phases I and II using anti-IL-12/23p40 antibodies (ustekinumab) in MS patients received conflicting results [134, 135]. ABT-874 is another monoclonal anti-IL-12/23 antibody, which exhibits similar safety in ustekinumab patients whereas efficacy for disease is lacking [136].

## 6. Dendritic Cells as a New Target for MS Treatment

The current treatment options for MS can affect DC function and phenotype. Although there is no DC-based drug for MS, there are multiple DC-based immunotherapies for EAE, a traditional animal model of MS. Tolerogenic DCs can be induced by multiple pathways. As previously described, tolerogenic DCs are able to induce periphery tolerance and alleviate the symptom of MS/EAE. These tolerogenic DCs have the therapeutic potentials for MS.

### 6.1. Estrogen

The relapse incidence of MS patients is lower during pregnancy, which suggests that estrogen may have a great effect on the pathogenesis of MS (Table 3). The following reports indicate that estrogen has immune modulation abilities. Murine treatment with estrogen decreased or even diminished EAE clinical symptoms [137, 138]. Further studies showed that the inhibition of estrogen on EAE was associated with DCs function [139]. Mice treated with estrogen have lower frequency of DCs migrating into the CNS of EAE [139]; the frequency of DCs producing IFN-*γ* and TNF-*α* was also reduced in spleens of EAE mice treated with estrogen [139]. Mature DCs treated with estrogen had a lower capacity for antigen presentation; moreover, secretion of proinflammatory cytokines was also inhibited* in vitro *[139, 140]. However, estrogen does not affect the expression of costimulatory molecules on DCs. Estrogen activates DCs and induces the expression of IDO, which inhibits T cell responses and reduces the production of both Th1 and Th2 cytokines [141]. In addition, DCs treated with estrogen (estrogen-DCs) showed tolerogenic properties, that is, reducing the infiltration of macrophages into the CNS and inhibiting T-cell proliferation in rats with EAE [141]. In MS patients, estrogen upregulates IDO expression on DCs and inhibits both Th1 and Th2 cytokines secretion [142]. Estrogen exerts its immune modulation role through its receptors *α* and *β* on DCs [143]. Although estrogen can relieve the symptoms of MS and reduce the incidence of relapses, it is not commonly used in clinic partly because of its side effects.

### 6.2. Vitamin D

**Figure 4:**
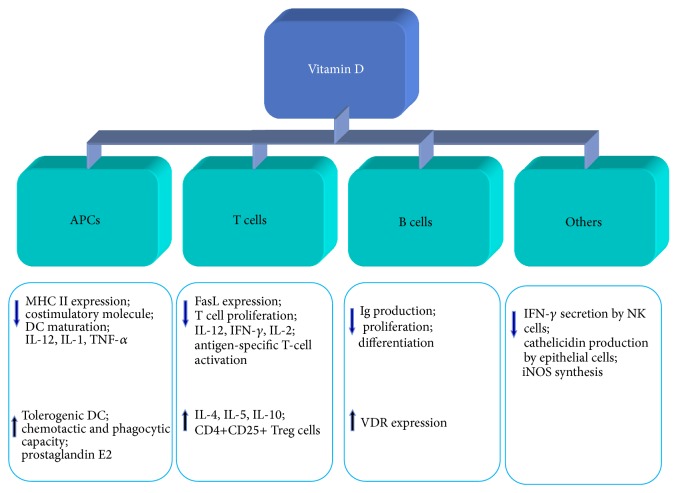
Effects of vitamin D on the immune system and immune responses. Vitamin D affects the immune responses including modulation of antigen-presenting cells (APCs), B, T, and NK cells. ↑ denotes induction or upregulation; ↓ denotes inhibition or downregulation. DCs: dendritic cells; iNOS: inducible nitric oxide synthase; IL: interleukin; IFN-*γ*: interferon gamma; Ig: immunoglobulin; M*φ*: macrophage; MHC: main histocompatibility complex; NK cells: natural killer cells; TNF-*α*: tumor necrosis factor alpha; VDR: vitamin D receptor.

The prevalence of MS increases as the latitude increases suggesting that the deficiency of sunshine increases the risk of MS [144]. It has been clearly established that vitamin D deficiency is a potential risk factor for MS [145], since the levels of vitamin D are lower in MS patients than in healthy subjects. Moreover, levels of vitamin D in MS patients suffering from relapse were lower than those during the remission stage [146]. Vitamin D plays an immunomodulatory role by its interaction with the vitamin D receptor (VDR), which is expressed on lymphocytes. 1, 25-dihydroxy vitamin D_3_ (1,25(OH)_2_D_3_) is the active form of vitamin D* in vivo*. The immunomodulatory function of vitamin D is carried out through several pathways [147] (Figure 4). 1,25(OH)_2_D_3_ *ex vivo *inhibits proliferation of MBP-specific T cells and increases the number of Tregs from MS patients [146]. Despite the effect on T cells, vitamin D also acts on DCs because of its immunomodulatory ability (Table 3).* In vitro*, 1,25(OH)_2_D_3_ can partially block the GM-CSF and IL-4-driven differentiation of monocytes into DCs [148]. When DCs, which differentiate from human monocytes in the presence of GM-CSF and IL-4* in vitro*, were treated with 1,25(OH)_2_D_3_, their capacity to mature into APCs was inhibited [148]. Likewise, when monocyte-derived DCs from RRMS patients were treated with 1,25(OH)_2_D_3_ the DCs exhibited an immature phenotype and the secretion of IL-12p40 was inhibited while the secretion of CCL2 increased [149]. The immunomodulatory role of vitamin D is partly via its ability to inhibit the differentiation and maturation of DCs and to generate tolerogenic DCs [150]. Tolerogenic DCs induced by 1,25(OH)_2_D_3_ resulted in stable antigen-specific hyporesponsiveness in myelin-reactive T cells from RRMS patients when stimulated with myelin peptide [150]. Vitamin D-induced tolerogenic DCs showed a significant increase in STAT3 and IDO expression and adoptive transfer of tolerogenic DCs significantly reduced the severity of EAE [151]. All these studies demonstrated the vitamin D-induced tolerogenic DCs have potential immunotherapy value. In recent years, the use of vitamin D_3_ supplementation to prevent MS or to slow disease progression is under extensive investigation. Vitamin D_3_ supplementation appeared beneficial to MS patients to some extent. Burton et al. found that the immunological parameters of MS patients taking vitamin D_3_ supplements are altered [152]. MS patients treated with vitamin D_3_ have fewer relapse events and more persistent reduction in T-cell proliferation compared to controls [152]. Another study indicated that there were no significant changes in clinical or radiological parameters of MS patients following short-term treatment with vitamin D_3_ supplementation, but the levels of cell proliferation were decreased [153]. It is noteworthy that the side effects of vitamin D_3_ should be taken into consideration if it is used as the treatment of MS.

### 6.3. Cytokines

Cytokines have been evidenced to participate in the pathogenesis of MS and can be divided into two types according to their function: proinflammatory and anti-inflammatory cytokines. The former promotes the process of demyelination and axonal damage, aggravating the severity of MS, while the latter promotes the inflammation subsided and disease recovery in MS. DCs are able to secrete both types of cytokines depending on the different subtypes and maturation stages. Herein we summarize the cytokines that are secreted by DCs and that may have potentials for MS treatment (Table 4).

#### 6.3.1. Interleukin- (IL-) 10

IL-10 is primarily produced by monocytes, macrophages, and different T cell subsets with the capacity to inhibit immune responses and induce immune tolerance. IL-10 can inhibit Th1 cells via multiple mechanisms. In addition, IL-10 can induce type 1 regulatory T cells (Tr1), which are essential in peripheral immune tolerance [154]. The main producers of IL-10 are Th2 cells, Tregs, and some types of DCs [155].

IL-10 exerts a “beneficial” role in MS by suppressing the autoimmune response through inhibiting self-antigen-specific T cells and inducting Tregs. IL-10 also could affect the function of DCs in MS.* In vitro*, DCs (monocyte-derived from MS patients) treated with IL-10 induced the production of IL-4 and IL-10 by autologous lymphocytes, while cDCs derived from MS and exposed to IL-10 became resistant to maturation induced by LPS [156]. The studies to date indicate that IL-10 is an important molecule that interferes with the maturation of DCs and induces tolerogenic DCs. Similarly, IL-10-treated DCs exhibited transient maturation, which expressed low level of IL-12 and failed to stimulate T cell proliferation both* in vivo* and* in vitro*. Interaction with IL-10-treated DCs rendered antigen-specific T cells unresponsive to subsequent challenges and adoptive transfer IL-10-treated DCs reduced the severity of EAE [157]. Recently, treatment with IL-10 gene-transfected mature DCs was found to be effective in suppressing EAE [158]. Gene therapy has not been applied in MS patients due to several reasons, yet a newly identified subset of DCs, so called DC-10, provides us with new perspectives. Unlike immature DCs and IL-10-treated DCs, DC-10 is a tolerogenic DC that has a stable mature phenotype due to its high expression of HLA-II and costimulatory molecules. DC-10 secretes high level of IL-10, together with the tolerogenic molecule HLA-G and the immunoglobulin-like transcript 4 inducer type 1 Tregs, which play an important role in peripheral tolerance [159]. DC-10 can differentiate from peripheral blood monocytes in the presence of GM-CSF, IL-4, and IL-10 [160]. Inducing the generation of DC-10 or IL-10-treated DCs* in vitro* then injecting them back into MS patients may be a potential therapeutic method in MS. However, it is necessary to conduct animal experiments and clinical trials to determine the safety and efficacy of this therapeutic alternative.

#### 6.3.2. TGF-*β*

Transforming growth factor- (TGF-) *β* is another anti-inflammatory cytokine. Nasal administration of low-dose TGF-*β*1 inhibited the development and relapse of protracted-relapsing EAE (PR-EAE) in DA rats [161]. Further studies showed that TGF-*β*1-induced suppression of PR-EAE is associated with apoptosis of CD4+ T cells induced by DC-derived nitric oxide [162]. TGF-*β*1-treated DCs exhibited the characteristics of immature or tolerogenic DCs, which had a lower capacity to stimulate T cells. Additionally, TGF-*β*1-treated DCs are potential therapeutic in Lewis rat with EAE [163]. TGF-*β* is necessary for the conversion of Th0 cells into Th17 cells. Integrin *α*v*β*8 expression on DCs can activate TGF-*β* and is critical in the differentiation of Th17 cells [164]. More importantly, integrin *α*v*β*8 on DCs is necessary for the induction of EAE, while mice lack of integrin *α*v*β*8 showed nearly complete protection from EAE [165]. When *α*v integrin expression on DCs was inhibited following drug treatment, TGF-*β* activation was blocked and Th17 generation was suppressed, which conferred protection from EAE in mice [165]. Therefore, TGF-*β* may have a potential therapeutic role in MS by two ways. Firstly, DCs treated with TGF-*β* can induce tolerogenic DCs, which could be used for cell therapy in MS. Secondly, drugs can be designed to target *α*v integrin on DCs to inhibit Th17 differentiation, and thus it achieves therapeutic purposes in MS.

#### 6.3.3. IL-12/IL-23

Besides, both IL-12 and IL-23 are proinflammatory cytokines secreted by APCs and can induce encephalitogenic T cells. IL-12 induces naive T cells differentiation into Th1 cells, while IL-23 is associated with the differentiation of Th17 cells [166]. IL-12 and IL-23 have the common subunit IL-12/23p40. Anti-IL-12/23p40 antibody significantly ameliorated EAE in rodents [130, 131] and non-human primates [132, 133]. Unfortunately, clinical trials in phases I and II using antibody against IL-12/23p40 (ustekinumab) in MS patients received conflicting results [134, 135]. ABT-874 is another monoclonal anti-IL-12/23 antibody, which exhibits similar safety as ustekinumab patients with lack of efficacy for disease [136].

#### 6.3.4. IL-17, IL-21, and IL-22

The IL-17 family includes six family members, namely, IL-17 A-F. In all of these family members, IL-17A is more critical in EAE than the others. The development of EAE IL-17A knock-out mice was significantly suppressed [167]. Neutralizing antibodies against IL-17A also ameliorated EAE symptoms [168]. Although further studies showed that IL-17A and IL-17F may only marginally contribute to the development of EAE [169], most researchers believed that IL-17A is dispensable for EAE induction. IL-17 gene polymorphism was found in all patients with each subtype of MS [170]. These findings imply that IL-17 may exert important roles in MS. Monoclonal antibodies against IL-17A have passed through phase II clinical trials. Th17 can secrete IL-21 and IL-22 as well. Mice deficient in IL-22, IL-21, and IL-21 receptors were all susceptible to EAE induction [171–173]. In IL-21 and IL-21 receptor knock-out mice, Th17 development and recruitment to CNS were normal [173]. These findings indicate that IL-21 and IL-22 are not effector cytokines in EAE. During the induction and peak phases of EAE, however, the levels of IL-22 were elevated [174]. More importantly, IL-22 was elevated in the serum of patients with MS [175].

#### 6.3.5. IL-1*β*

IL-1*β* is an important pathogenic cytokine in EAE. IL-1*β* receptor knock-out mice have a significant reduction in disease severity during EAE [176]. IL-1*β* receptor knock-out mice show significant decrease in VCAM-1 expression and diminished leukocytes infiltration in the spinal cord in animals challenged with EAE [177]. Th17 differentiation was also affected [177]. In brain lesions of patients with MS, high levels of IL-1*β* were found [178].

#### 6.3.6. GM-CSF

GM-CSF is IL-23 driven cytokines in EAE and is required for Th17 to become encephalitogenic cells [179]. Moreover, GM-CSF would enhance IL-23 secretion by APCs [179]. Autoreactive T helper cells specifically lacking GM-CSF failed to induce EAE despite expression of IL-17A or IFN-*γ*, whereas GM-CSF secretion by IFN*γ* ^−/−^ IL-17A^−/−^ helper T cells was sufficient to induce EAE [180]. During the disease effector phase, GM-CSF sustained neuroinflammation via myeloid cells that infiltrated the CNS [180]. Langerin (+) CD103 (+) DCs play a key role in EAE, which activate encephalitogenic T cells in the periphery prior to other DCs. Their accumulation in the skin and peripheral lymph nodes is dependent on GM-CSF [181]. GM-CSF is also critical in either initiation or plateau stages of EAE. Phase II clinical trials on monoclone antibodies against GM-CSF have been finished.

#### 6.3.7. TNF-*α*

There are two kinds of TNF-*α*, membrane bound TNF-*α* (mTNF-*α*) and soluble form TNF-*α*. TNFR1 is widely expressed and may bind two kinds of TNF, while TNFR2 is expressed on lymphocytes and binds to soluble membrane bound TNF-*α* [182]. TNF-*α* is a proinflammatory cytokine, so mice treated with antibody against TNF-*α* were found resistant to EAE [183]. Mice deficient in TNFR1 or TNFR1/TNFR2 were resistant to EAE, whereas mice deficient in TNFR2 exhibited severe EAE symptoms [184, 185]. These studies suggested that mTNF signaling with TNFR2 has a protection role [186]. This protection role could explain why plenty of patients treated with anti-TNF-*α* agent in clinical trials result in disease aggravation [187].

#### 6.3.8. IL-6

IL-6 is a proinflammatory cytokine; together with TGF-*β*, IL-6 promotes Th17 differentiation. IL-6 knock-out mice are completely resistant to EAE induction [188]. IL-6 receptor blockade prevents EAE induction through inhibiting Th17 differentiation [189]. Recently researchers have found that the key cells that are required to produce IL-6 to drive EAE are DCs. At the initial stage of EAE, DCs derived IL-6 is critical for T cell activation and EAE induction [190]. In MS patients, IL-6 was found in the brain. This suggests that IL-6 participates in the pathogenesis of MS [191]. Tocilizumab is a blocking antibody against the IL-6 receptor. In clinical trials, beneficial effects have been shown on rheumatoid arthritis. There has been no clinical trial on this drug as yet, whereas a recent case report showed that treatment with tocilizumab has positive effects on patients with neuromyelitis optica [192]. Thus, tocilizumab would be an effective therapy for CNS demyelinating disorders.

## 7. Will DCs Be a Therapeutic Tool for MS in Future?

As previously mentioned, DCs are important in the pathogenesis of MS and current options for MS directly or indirectly affect DCs function. Recently, cell therapy has become hot spot in cancer, transplant, and autoimmune diseases. As its double role in MS, DCs would be an ideal tool for cell therapy. At present, it is hard to achieve in patients with MS, while in basic research, researchers used kinds of methods modifying DCs* in vitro* and then adoptive transfer of the modified DCs to EAE model. Early in 2002 Menges et al. showed that repetitive injections of TNF*α* matured DCs plus peptide protected mice from EAE induction [193]. Hirata et al. used genetic skills modified DCs, making DC presenting MOG peptide in the context of MHC class II molecules and simultaneously expressing TRAIL or PD-L1. Mice treated with such modified DCs had ameliorated MOG peptide-induced EAE scores; the T cell response to MOG and cell infiltration in spinal cord were also reduced [194]. Treatment of DCs with chloroquine (CQ) induced tolerogenic DCs, and adoptive transfer CQ-DCs to EAE mice would reduce the clinical manifestation of the disease [195]. Moreover, Zhou et al. recently find either intravenous transfer of LPS-treated DCs or apoptotic cell-treated DCs blocking EAE [196, 197].

pDC is another subset of DCs, the pathogenesis roles of which on EAE are interesting. At first, researchers found that CNS pDCs suppressed CNS mDC-driven production of IL-17, IFN-*γ*, and IL-10 in an IDO-independent manner. They concluded that pDCs play a critical regulatory role in negatively regulating pathogenic CNS CD4(+) T cell responses [56]. Then, Isaksson et al. used anti-PDCA1 antibodies to deplete pDCs at different stages of EAE and found that pDC had different roles in different stages of EAE. When pDCs were depleted prior to MOG induction, pDC-depleted mice developed less severe EAE, which implied that pDCs have a promoting role in the initiation phase of EAE [198]. When pDCs were deleted a week after the immunization, pDC-depleted mice developed more severe symptoms, demonstrating a protecting role of pDCs in EAE [198]. After EAE is induced, pDCs are recruited to lymph nodes where MHC-II-dependent myelin-Ag-specific contacts with CD4+ T cells are established. These interactions may promote the selective expansion of natural Tregs to inhibit the autoimmune T cell response [57]. Taken together, pDCs' protective roles in EAE enlighten us that pDCs would be one of effective therapeutic options for MS. For instance, we can increase the amount of pDCs in CNS to suppress the pathogenic CNS CD4+ T cell responses. We can also transfer myelin-Ag-loaded pDCs to stimulate Treg induction by pDCs, which may eventually benefit MS patients [57].

## 8. Conclusion

In summary, we summarized the role of DCs in the pathogenesis of MS and current therapeutic options for MS affected the subset and function of DCs. The development of novel therapy to target DCs is therefore essential. Drugs and cytokines that have potential therapeutic effects on MS may be an alternative option. Targeted therapies against detrimental DCs in MS would be promising in the future.

## Figures and Tables

**Table 1:** Human dendritic cell subsets.

	Conventional DCs (cDCs)		Plasmacytoid DC (pDC)
	CD1c+DC	CD141+DC	
Specific marker	Lin−HLA-DR+CD11C+CD1c+	Lin−HLA-DR+CD11C+CD141+	Lin−HLA-DR+CD123+CD303+CD304+
C-type lectin	BDCA-1	BDCA-3	BDCA-2, BDCA-4
TLR expression	TLR1-5, TLR6, TLR8	TLR3	TLR7, TLR9
Cytokines secreted	IL-8, TNF-*α*, IL-6, CCL3 CCL4	IL-12p70, IFN-*β*	Type I interferon, TNF-*α*, IL-6
Role	Th2 type response Immune modulation	Th1 type response cross-present exogenous antigen	Antiviral inflammation Peripheral immune tolerance

**Table 2:** Effects of current therapeutic options on DCs in multiple sclerosis.

Drugs	Subset and quantity of DCs	Cytokines secreted	Surface markers
Corticosteroids	pDC↓		
	cDC↓		

Interferon-*β*	cDC↓	IL-12↓	CCR7↓
		IL-1*β*↓	MMP-9↓
		IL-23↓	MHC-II↓
			BDCA-2↓
	pDC↑	IL-10↑	CD83↑
	pDC1/pDC2↑	IL-27↑	B7-H1↑

Glatiramer acetate		IL-17↓	HLA-DR↓
		ROR-*γ*↓	CD86↓
		IL-12↓	CD40↓
		MIP-1*α*↓	4-1BBL↓
		IP-10↓	
		IL-10↑	

Natalizumab	CD209+DC↓		VLA-4↓

Fingolimod		IL-12↓	
		IL-10↑	
Teriflunomide		IL-6↓	
		IL-8↓ MCP-1↓	
Dimethyl fumarate		IL-12↓ IL-23↓	
		IL-10↑	

Laquinimod^*※*^	CD1c+DC↓ CD303+DC↓		CD86↓

Daclizumab^*※*^		IL-12↓ IL-1↓ TNF-*α*↓ IL-6↓ IFN-*γ*↓	
		IL-10↑	

^*※*^laquinimod and daclizumab are under phase III clinical trial.

**Table 3:** Factors associated with MS and DCs.

	Immunological effect	Effect on MS/EAE
Vitamin D	Induces Tregs and tolerogenic DCs	Reduces relapses of MS and changes clinical parameters of MS

Estrogen	Inhibits Th1 and Th2 cytokines secretion; induces tolerogenic DCs	Relieves symptoms of MS and reduces incidence of MS

**Table 4:** Cytokines associated with DC and MS.

Cytokines	Immunological role in EAE/MS	Influence on MS/EAE
IL-10	Inducing t-DC Inducing Tr1	Reducing EAE through inhibiting self-antigen-specific T cells and inducting Tregs

TGF-*β*	Inducing th17 and Treg	Adoptive transfer of tolerogenic DCs alleviates EAE

IL-12/IL-23	Inducing Th1/Th17 differentiation	Antibody alleviates EAE but not MS patient

IL-17/IL-21/IL-22	The main effector cytokines of Th17	IL-17 is an effector cytokine in EAE/MS Antibodies against IL-17 may play a therapeutic role

IL-1*β*	Promoting Th17 differentiation	Being essential for EAE

GM-CSF	Promoting DCs maturation and Th17 differentiation	Initiating or sustaining EAE Antibodies against GM-CSF may be an alternative for MS

IL-6	Promoting Th17 differentiation	Being essential for EAE, participating MS pathogenesis; antibodies against IL-6 would be effective in MS.

TNF-*α*	Binding to TNFR1 to induce cell apoptosis Binding to TNFR2 to maintain cell survival	TNFR1 mediates demyelination and TNFR2 remyelination
